# Rare histologic transformation of a *CTNNB1* (β-catenin) mutated prostate cancer with aggressive clinical course

**DOI:** 10.1186/s13000-024-01511-3

**Published:** 2024-06-21

**Authors:** Dilara Akhoundova, Stefanie Fischer, Joanna Triscott, Marika Lehner, Phillip Thienger, Sina Maletti, Muriel Jacquet, Dinda S.H. Lubis, Lukas Bubendorf, Wolfram Jochum, Mark A. Rubin

**Affiliations:** 1 Department for BioMedical Research, University of Bern, Bern, 3008 Switzerland; 2 Department of Medical Oncology, Inselspital, University Hospital of Bern, Bern, 3010 Switzerland; 3 Department of Medical Oncology and Hematology, Cantonal Hospital St. Gallen, St. Gallen, 9007 Switzerland; 4 Institute of Medical Genetics and Pathology, University Hospital of Basel, Basel, 4031 Switzerland; 5 Institute of Pathology, Cantonal Hospital St. Gallen, St. Gallen, 9007 Switzerland; 6 Bern Center for Precision Medicine, Inselspital, University Hospital of Bern, Bern, 3008 Switzerland

**Keywords:** Prostate cancer, Metastatic castration-resistant prostate cancer (mCRPC), *CTNNB1* mutation, Wnt/β-catenin pathway, Histologic transformation, Targeted treatment, CK1 inhibitors, Tankyrase inhibitors

## Abstract

**Background:**

*Catenin (Cadherin-Associated Protein), Beta 1* (*CTNNB1)* genomic alterations are rare in prostate cancer (PCa). Gain-of-function mutations lead to overexpression of β-catenin, with consequent hyperactivation of the Wnt/β-catenin signaling pathway, implicated in PCa progression and treatment resistance. To date, successful targeted treatment options for Wnt/β-catenin - driven PCa are lacking.

**Methods:**

We report a rare histologic transformation of a *CTNNB1* (β-catenin) mutated metastatic castration resistant prostate cancer (mCRPC), clinically characterized by highly aggressive disease course. We histologically and molecularly characterized the liver metastatic tumor samples, as well as successfully generated patient-derived organoids (PDOs) and patient-derived xenograft (PDX) from a liver metastasis. We used the generated cell models for further molecular characterization and drug response assays.

**Results:**

Immunohistochemistry of liver metastatic biopsies and PDX tumor showed lack of expression of typical PCa (e.g., AR, PSA, PSAP, ERG) or neuroendocrine markers (synaptophysin), compatible with double-negative CRPC, but was positive for nuclear β-catenin expression, keratin 7 and 34βE12. *ERG* rearrangement was confirmed by fluorescent in situ hybridization (FISH). Drug response assays confirmed, in line with the clinical disease course, lack of sensitivity to common drugs used in mCRPC (e.g., enzalutamide, docetaxel). The casein kinase 1 (CK1) inhibitor IC261 and the tankyrase 1/2 inhibitor G700-LK showed modest activity. Moreover, despite harbouring a *CTNNB1* mutation, PDOs were largely insensitive to SMARCA2/4- targeting PROTAC degraders and inhibitor.

**Conclusions:**

The reported *CTNNB1-mutated* mCRPC case highlights the potential challenges of double-negative CRPC diagnosis and underlines the relevance of further translational research to enable successful targeted treatment of rare molecular subtypes of mCRPC.

**Supplementary Information:**

The online version contains supplementary material available at 10.1186/s13000-024-01511-3.

## Background

*Catenin (Cadherin-Associated Protein), Beta 1* (*CTNNB1)* genomic alterations are rare in prostate cancer (PCa), being found in ∼3–5% of the cases [1, 2]. Gain-of-function *CTNNB1* mutations lead to nuclear overexpression of β-catenin, which is a core component of the canonical Wnt/β-catenin pathway [3]. β-catenin plays a relevant role in PCa carcinogenesis, disease progression and therapy resistance [1, 4–9]. An epigenomics and transcriptomics-based classification of metastatic castration resistant prostate cancer (mCRPC)-derived organoids, proposed four subgroups of mCRPC, including a Wnt-driven subtype [10]. Three out of four patient-derived organoids (PDOs) in the Wnt-driven subgroup harboured a missense hot spot mutation in *CTNNB1*, with concomitant increased messenger RNA (mRNA) expression [10]. Previous studies have shown that activation of the Wnt/β-catenin pathway confers resistance to treatment with androgen receptor (AR) pathway inhibitors (ARPIs) and chemotherapy with docetaxel [6, 11–16]. However, to date little is known about the biological disease course of PCa with Wnt//β-catenin pathway activation, and no molecularly targeted treatment strategies are available for these tumors. We report a case of rare histologic transformation of a *CTNNB1*-mutated metastatic PCa, clinically characterized by a fulminant disease course. We, moreover, generated a 3D PDO as well as a patient-derived xenograft (PDX) from patient’s liver metastatic biopsy, characterized them histologically and molecularly, as well as used the generated cellular models to assess response to several standard and experimental drugs.

## Methods

### Patient-derived organoids (PDOs)

3D organoids were derived following previously published protocols [17]. Briefly, fresh tumor tissue was mechanically and enzymatically dissociated using collagenase type II at a concentration of 5 mg/ml (Gibco™, catalog 17,101,015), supplemented with 10 µM of Y-27,632 (Selleck Chemicals, catalog S1049). Tumor tissue was incubated during 1 h in collagenase II in a 1.5 ml tube and at 37 °C on a shaker. Following enzymatic digestion, dissociated tumor tissue was washed in advanced DMEM/F12 (Gibco™, catalog 12,634,010) supplemented with GlutaMax (Gibco™, catalog 35,050,061), Hepes (Gibco™, catalog 15,630,056) and penicillin-streptomycin (Gibco™, catalog 15,140,122) (adDMEM/F12 +++) and centrifuged for 5 min at 250G and 4 °C. 1 ml of TrypLE Express (Gibco™, catalog 12,605,028) with 10 µM Y-27,632 was added to the pellet for further digestion during approximately 15 min at 37°. Mixture was repetitively (ca. every 5 min) pipetted up and down to ensure optimal dissociation. After washing with adDMEM/F12 +++, pellet was resuspended in undiluted ice-cold Matrigel and quickly placed as 40 µl Matrigel (VWR, catalog BDAA356239) droplets in the middle of a prewarmed 24-well cell culture plate (Corning, catalog 3526). To allow Matrigel solidification the plate was placed into cell culture incubator at 37° for 10–15 min. Afterwards, prewarmed human prostate cancer medium was added, and changed every 3–4 days [17]. Growth of derived PCa organoids was monitored and they were passaged 1:2 every 7 to 14 days.

### Patient-derived xenograft (PDX)

PDOs from passage 6 were used for PDX generation. 1.8 × 10^6^ viable cells were injected subcutaneously (sc) in a 2-weeks old NOD scid gamma (NSG) male mouse. The mouse was regularly assessed for sc tumor growth (initially once weekly, and 2 times/week from detection of palpable tumor). Tumor would be allowed to grow to a maximum of 1 cm ^3^. All experiments were performed in agreement with local laws and regulations.

### Immunohistochemistry (IHC)

IHC stainings were performed at the Translation Research Unit (TRU) of the Institute of Tissue Medicine and Pathology, University of Bern, as well as at the Institute of Pathology of the Cantonal Hospital of St. Gallen. Staining was performed on formalin-fixed paraffin embedded (FFPE) PDX tumor slides. Slides were stained with H&E, as well as antibodies against AR, PSA, PSAP, synaptophysin, ERG, keratin 7 (KRT7), 34βE12 and β-catenin. Following antibodies have been used: anti-AR (AR441, Cell Marque™, catalog 200 M-15, 1:100), anti-PSA (polyclonal rabbit, Dako, catalog A0562, 1:4000), anti-PSAP (PASE/4LJ, Dako, catalog M0792, 1: 2000), anti-synaptophysin (27G12, Novocastra, catalog NCL-L-SYNAP-299, 1:100), anti-ERG (EP111, Dako, catalog M73149, 1:50), anti-human KRT7 (OV-TL 12/30, Cell Marque™, catalog 307 M-96, 1: 800), anti-34βE12 (Dako, catalog M0630, 1: 200), β-catenin (Abcam, catalog ab35572, 1:2000).

### DNA sequencing

DNA was extracted from fresh-frozen liver metastatic tumor tissue with using the AllPrep DNA/RNA FFPE kit (Qiagen, catalog 80,234), and DNA concentration measured with Qubit (Thermo Fischer Scientific). Hybrid capture-based next-generation sequencing (NGS) was performed using the commercially available FoundationOne® CDx assay, which interrogates 324 genes for substitutions, indels and copy number variations, and 36 genes for rearrangements, as well as provides information on tumor mutational burden (TMB) and microsatellite instability status [18].

### Fluorescent in situ hybridization (FISH)

FISH for the detection of the *TMPRSS2/ERG* rearrangement was performed at the Institute of Pathology, University Hospital Basel, Switzerland, using the commercially available Zyto*Light® ERG*-dual color break apart probe (ZytoVision GmbH, Bremerhaven, Germany, catalog Z-2138-200), according to the manufacturer’s recommendations. Signals were counted in at least 50 tumour nuclei using an epifluorescence microscope (Axioplan 2 Imaging; Carl Zeiss, Oberkochen, Germany). FISH for *ERG* rearrangement was defined by the loss of one green signal (5′ probe) or as a separate green, and a separate orange signal in at least 15% of analyzed tumor cells.

### Drug response assays

For drug response assays, PCa organoids were enzymatically and mechanically dissociated, allocating 1000 cells/well in previously Matrigel-coated 6-well-plate (Corning, catalog 3516), and cultivated for 24 h before drug treatment. Cell viability read-out with CellTiter-Glo® 3D Cell Viability Assay (Promega, catalog G9683) was performed after 120 h of drug exposure. Drugs were tested in a range of concentrations 1nM -10 µM to determinate the IC50. Generated data were analyzed by GraphPad Prism®, and IC50 curves for tested drugs were generated using the log(inhibitor) vs. response curves. We assessed response to following drugs: enzalutamide (Selleckchem, catalog S1250), docetaxel (MedChem Express, catalog HY-B0011), CFI-402,257 (GLPBio, catalog GC18491), IC261 (MedChem Express, catalog HY-12,774), G007-LK (MedChem Express, catalog HY-12,438), iCRT14 (MedChem Express, catalog HY-166,665), RCM1 (Selleckchem, catalog S6898), AU15330 (synthesized by Genentech), A947 (synthesized by Genentech), FHD286 (Genentech), ACBI1 (Boehringer Ingelheim) and VZ185 (Boehringer Ingelheim).

## Results

### Clinical presentation

**Fig. 1:**
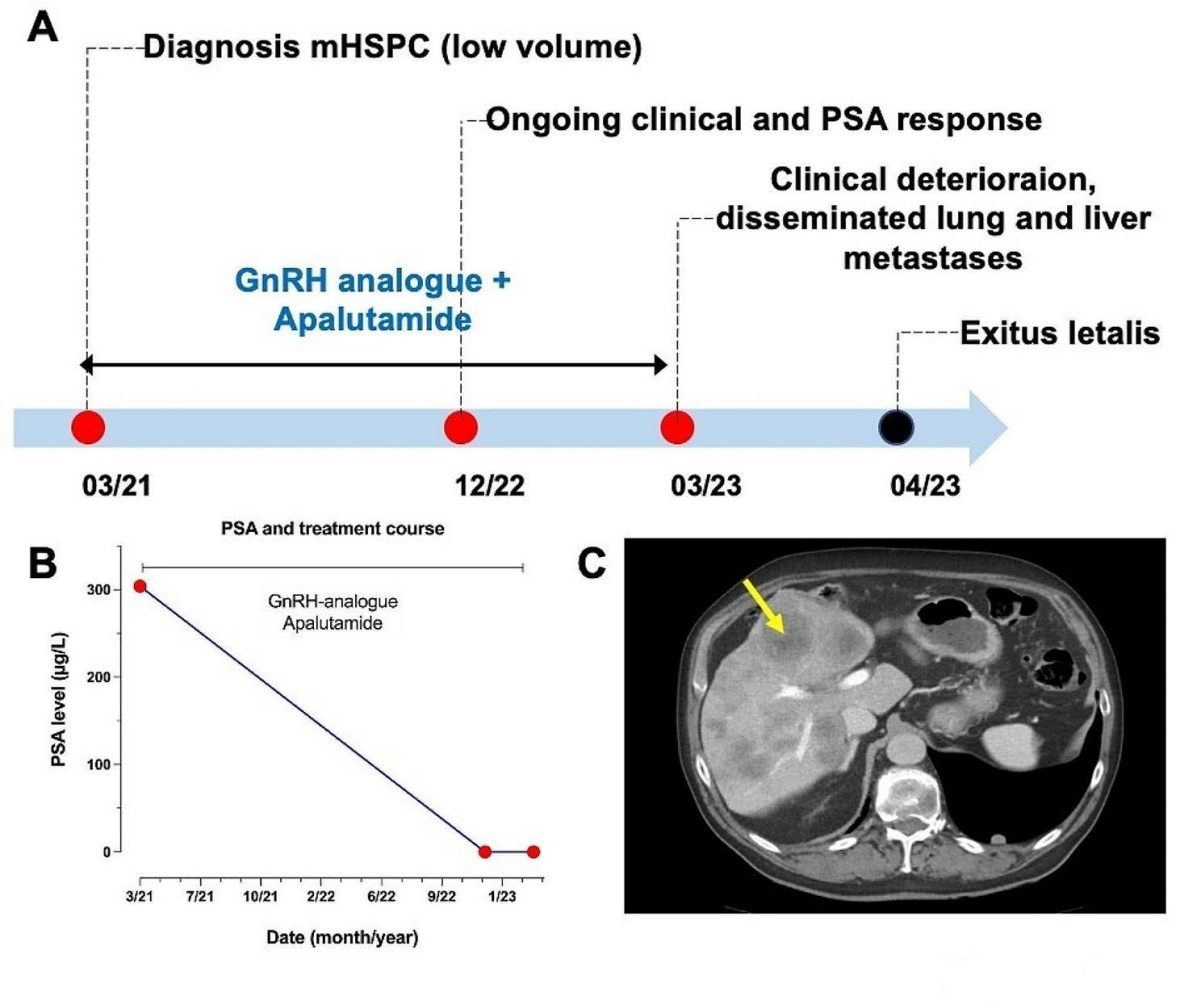
Schematic illustration of patient’s clinical disease course. (**A**) Timeline showing disease course and received treatment lines; (**B**) PSA levels over disease course; (**C**) Computed tomography image of liver metastatic spread (yellow arrow shows liver metastases). GnRH: gonadotropin-releasing hormone; mHSPC: metastatic hormone-sensitive prostate cancer; PSA: prostate-specific antigen

An 82-year-old patient was diagnosed with *de novo* metastatic hormone-sensitive prostate cancer. His previous medical history included hypertension, dyslipidemia, and atrial fibrillation. Biopsy of the prostate revealed an adenocarcinoma Gleason score (GS) 4 + 4 = 8 with pronounced cribriform growth pattern without neuroendocrine differentiation. The prostate-specific antigen (PSA) value at presentation was 304 µg/l. Conventional imaging with computer tomography (CT) of chest-abdomen and bone scan showed pelvic and abdominal lymph node metastases (M1a), with no signs of visceral or bone involvement. First-line treatment with a gonadotropin*-*releasing hormone (GnRH) analogue and apalutamide was initiated. Radiotherapy to the prostate given low volume disease analog STAMPEDE trial was evaluated but rejected by the patient [19]. Under first-line treatment with GnRH and apalutamide, the PSA value dropped down to 0.04 µg/l. Two years after treatment initiation the patient reported intense fatigue, dysuria, and peripheral edema of the lower limbs. Laboratory values revealed a grade 1 increase of transaminases, an alkaline phosphatase of 288U/l and a lactate dehydrogenase of 646 U/l, as well as a low PSA (0.1 µg/l) and elevated neuron-specific enolase values (236 µg/l). CT imaging revealed local progression of the primary tumor infiltrating bladder and rectum and new extensive metastatic spread to the lung and liver. Liver biopsy was performed to assess small-cell transformation, revealing a highly proliferative high-grade adenocarcinoma with prominent nucleoli; no neuroendocrine features were observed. Based on the working current classification of advanced PCa, this tumor fits into a class of double-negative CRPC expressing neither AR or neuroendocrine features (personal communication PCF Pathology working group). The patient presented rapid clinical deterioration complicated with sepsis and leading to death one month later (Fig. 1).

### Phenotypic and genomic characterization

**Fig. 2:**
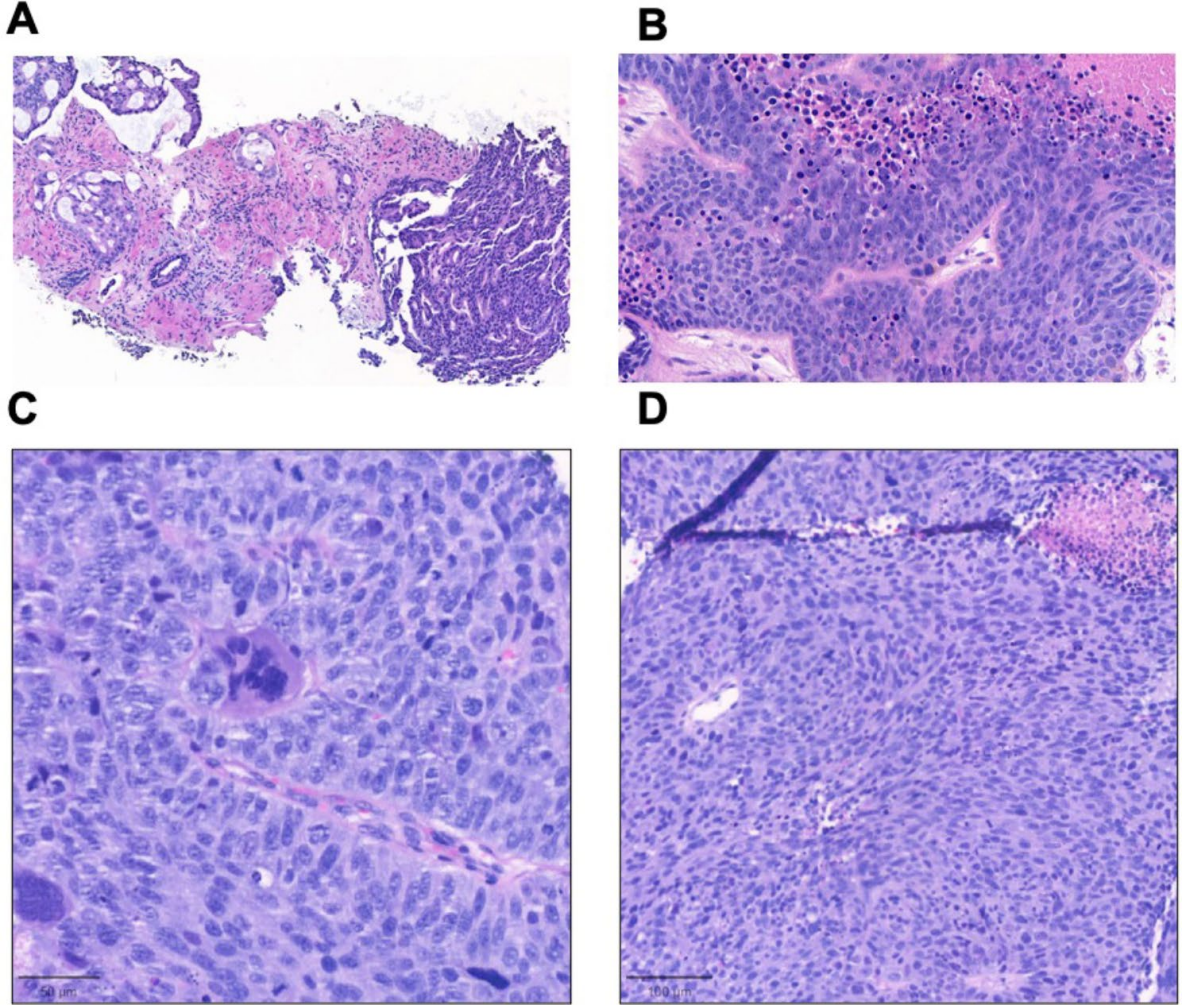
Hematoxylin and eosin (H&E) staining of (**A**) primary PCa tumor, (**B**) liver metastases and (**C**) patient-derived xenograft (PDX) tumor material. (**A**) Primary PCa tumor showing glandular formations with mucin; (**B**) Liver metastasis showing highly proliferative high-grade adenocarcinoma with prominent nucleoli; no neuroendocrine features are observed; (**C**) PDX tumor material showing highly proliferative neoplasia, histologically similar to the liver metastatic biopsy

**Fig. 3:**
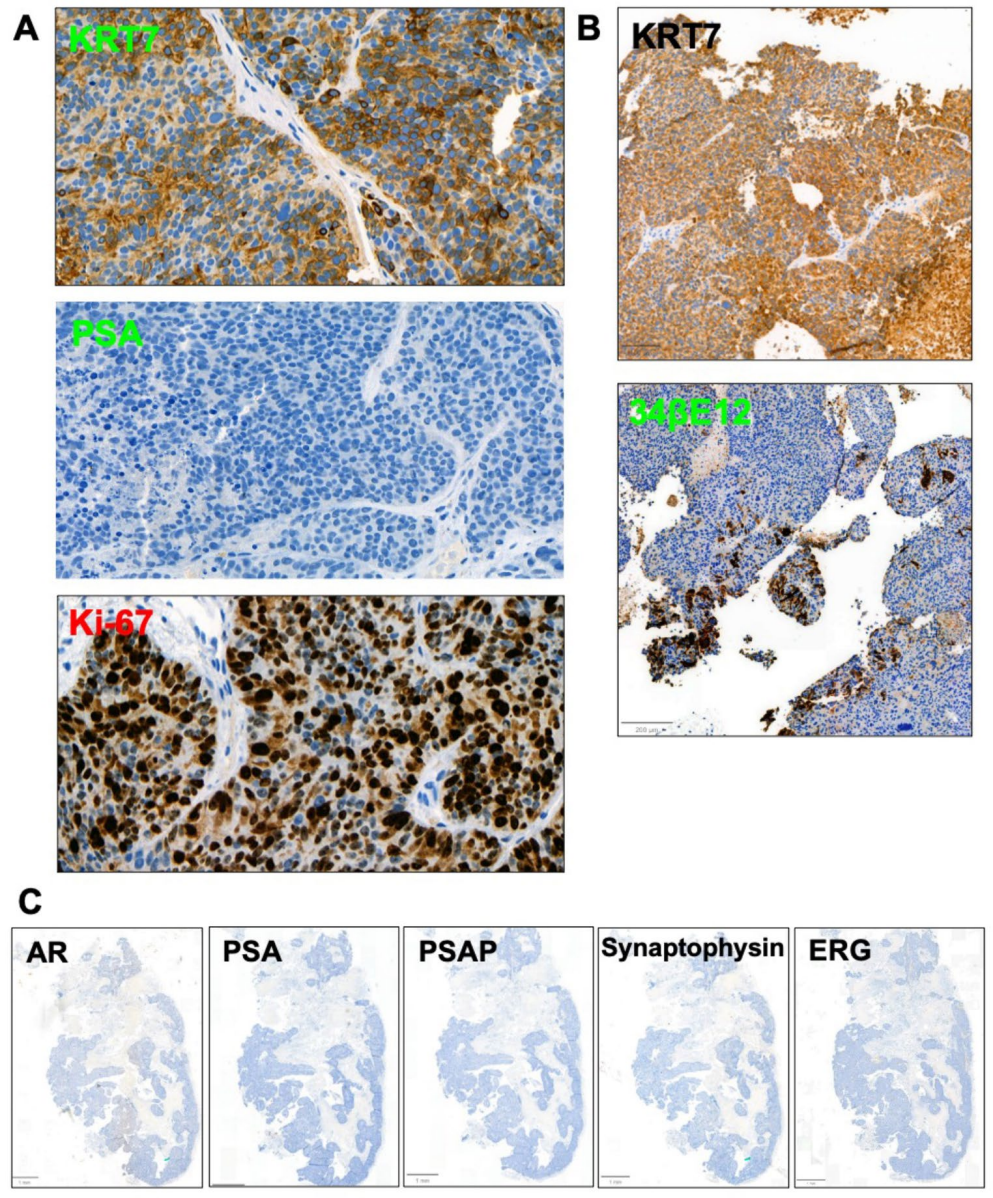
Immunohistochemistry (IHC) stainings of liver metastatic biopsy material and PDX histology slides. (**A**) Strongly positive IHC staining for KRT7, negative IHC for PSA and high Ki-67 index (90–95%), liver metastatic biopsies. (**B**) strongly positive IHC staining for KRT7 and focally positive for 34βE12, PDX tumor; (**C**) Negative IHC stainings on PDX tumor for AR, PSA PSAP, synaptophysin and ERG. 34βE12: cytokeratin 34βE12; AR: androgen receptor; KRT7: keratin 7; ERG: ETS-related gene; PSA: prostate-specific antigen; PSAP: prostate-specific acid phosphatase

**Fig. 4:**
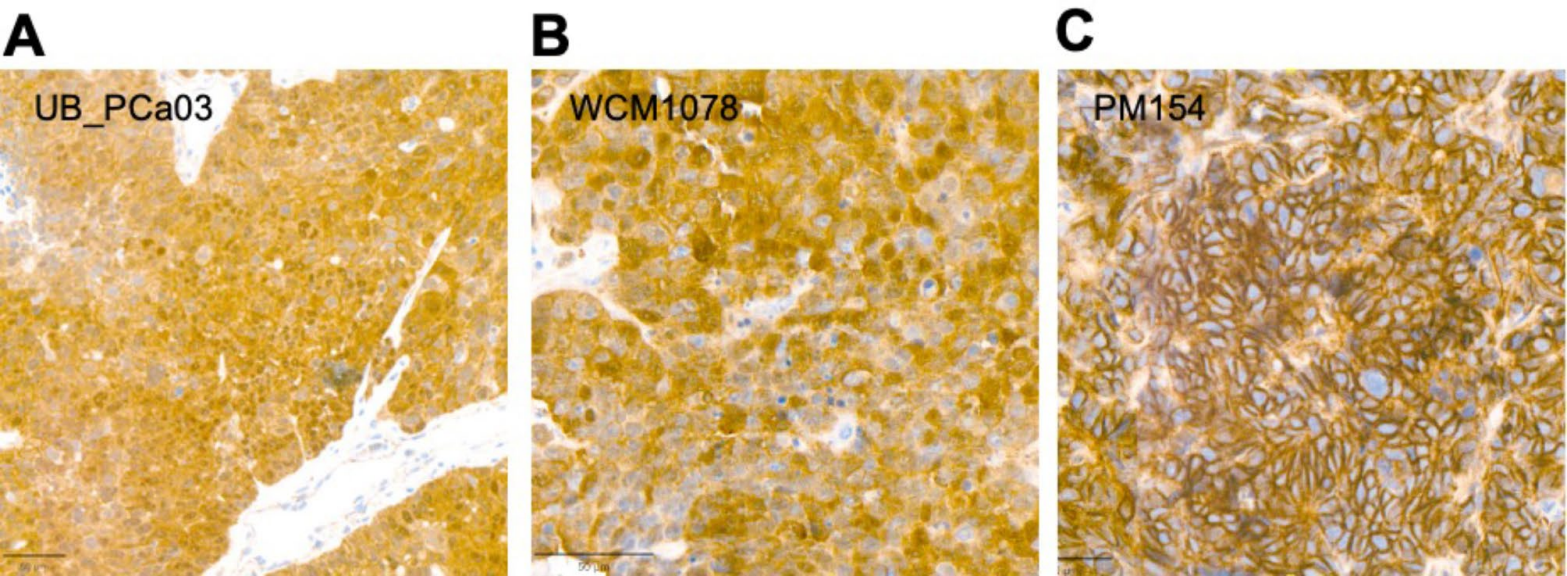
IHC staining for β-catenin in derived PDX (UB_PCa03) and two other Wnt-dependent established PDOs. Strong nuclear positivity for β-catenin staining is observed in the reported PDX UB-PCa03 (**A**), as well as in the established Wnt-dependent PDO WCM1078 (**B**). The neuroendocrine PDO PM154 (**C**) shows uniquely cytoplasmatic staining for β-catenin, as negative control

**Fig. 5:**
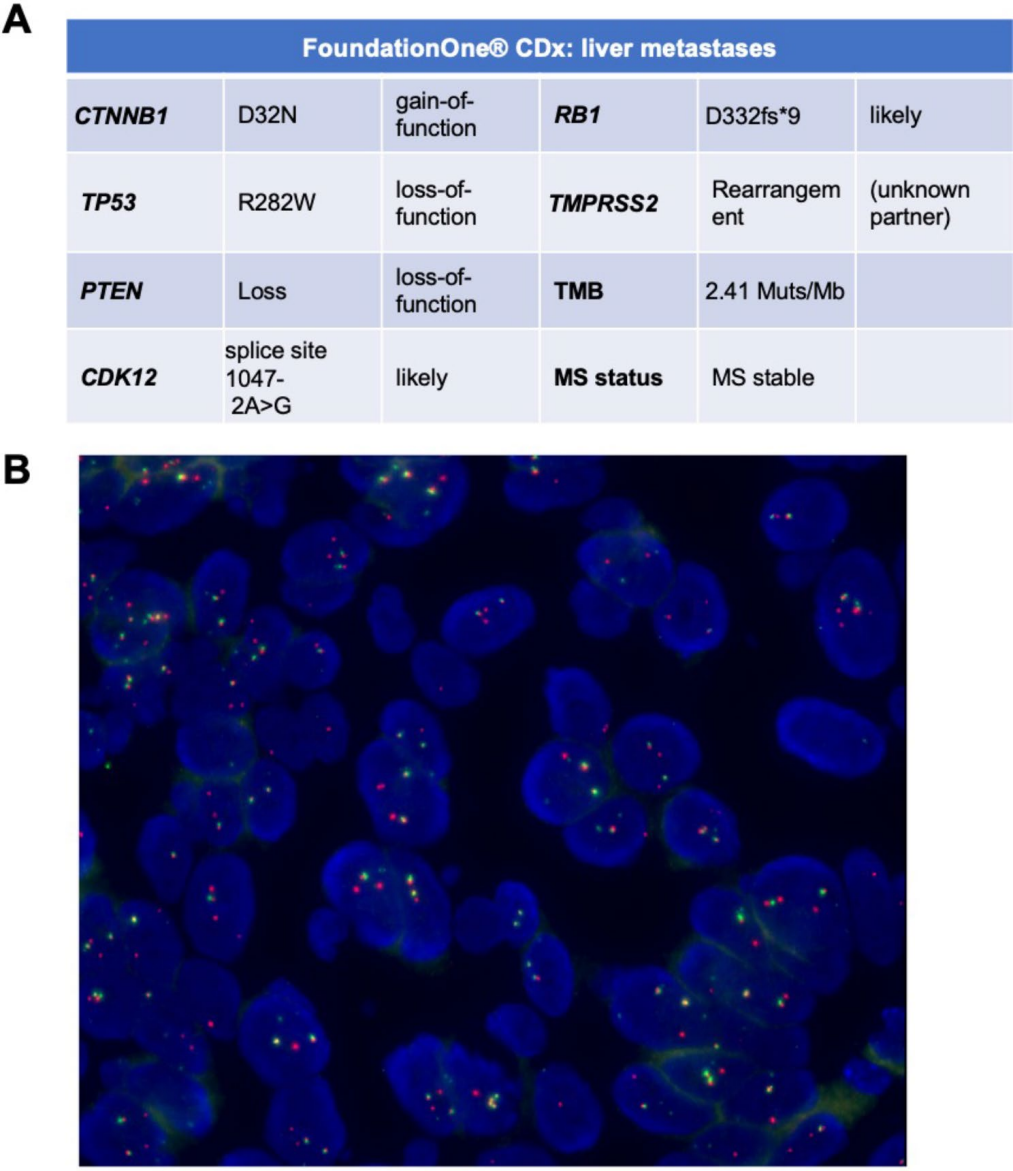
Next-generation sequencing (NGS) and fluorescent in situ hybridization (FISH) for *TMPRSS2/ERG* rearrangement. (**A**) NGS (FoundationOne® CDx) results from liver metastatic biopsy. (**B**). FISH confirming presence of the *ERG* rearrangement in 92% of the analyzed cells on PDX histologic material. MS: microsatellite; TMB: tumor mutational burden

While primary PCa tumor showed mucin-containing glandular formation, liver biopsy and PDX hematoxylin and eosin (H&E) slides showed high-grade solid carcinoma with prominent nucleoli, lack of cribriform growth pattern, absence of acid mucin and no evidence of neuroendocrine features (Fig. 2). The liver metastases and PDX tumor histology slides were negative for AR, PSA, PSAP and ERG protein expression; the tumor strongly expressed KRT7 and focally 34βE12 (Fig. 3). To help exclude a urothelial carcinoma keratin 34βE12 was performed, resulting in focal positivity (Fig. 3A-B) [20]. β-catenin IHC nuclear staining was strongly positive in the derived PDX (UB_PCa03), as well as in the established Wnt-dependent PCa PDO WCM1078 [10] (Fig. 4A-B). On the contrary, in the neuroendocrine PDO PM154, β-catenin staining was uniquely cytoplasmatic, acting as a negative control (Fig. 4C). Targeted DNA NGS (FoundationOne®CDx) performed from the liver metastatic biopsy material uncovered a hotspot *CTNNB1* gain-of-function mutation in exon 2 (94G > A, D32N), as well as a loss-of-function mutation in *TP53* (R282W), along with *PTEN* loss and a *TMPRSS2* rearrangement (with unclear partner). The tumor molecular burden was low (2.4 mutations/megabase) and the microsatellite status stable (Fig. 5A, Suppl. Table 1). The presence of an *ERG* rearrangement was confirmed by FISH on the PDX histology slides, showing a split signal in 92% of the analyzed tumor cells (Fig. 5B).

### Patient-derived organoids and xenografts

**Fig. 6:**
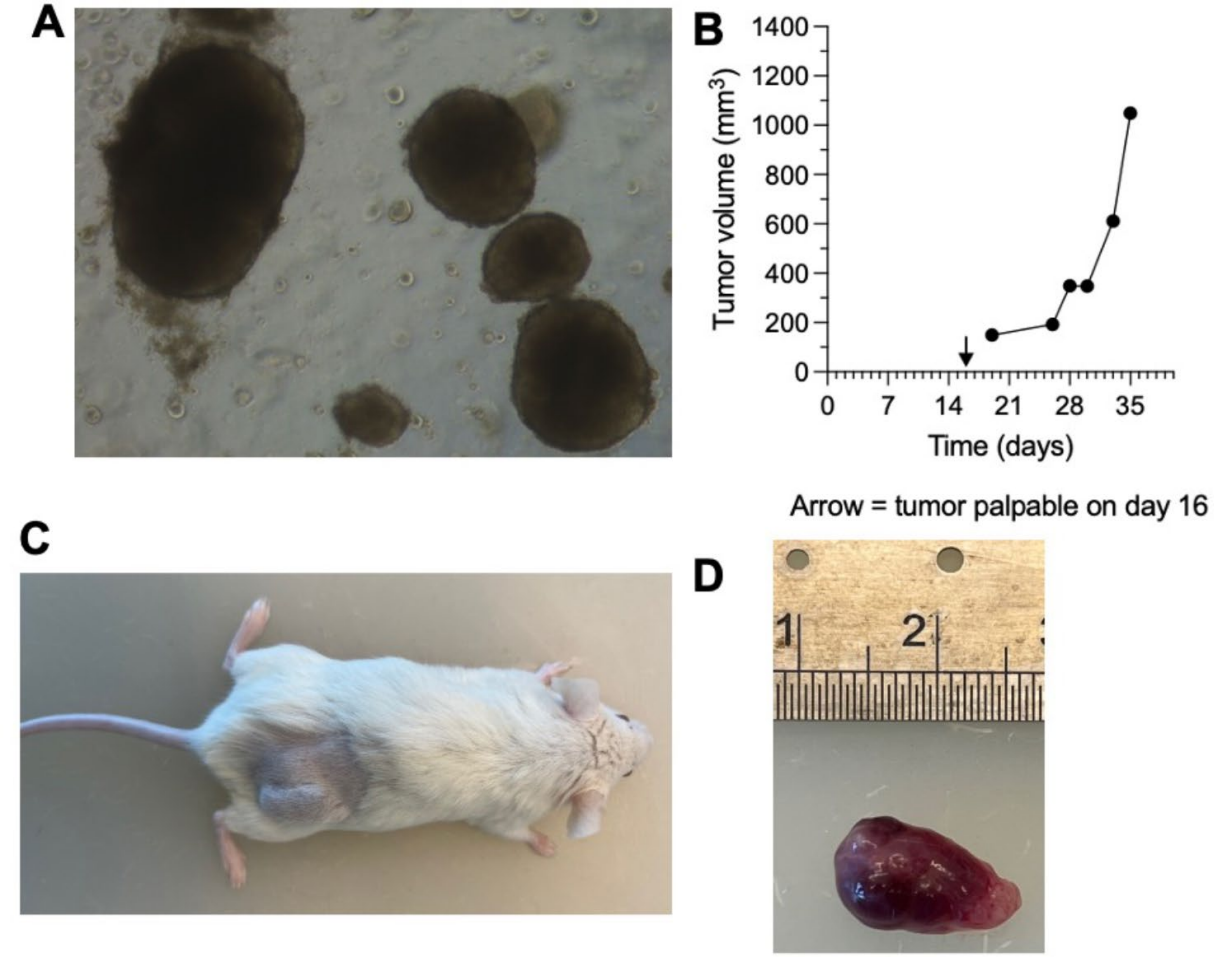
Patient-derived organoids (PDOs) and xenograft (PDX). (**A**) PDO derived from liver metastatic biopsies, passage 6, day + 10, objective 5x; (**B**) PDX growth curve: palpable subcutaneous tumor was first detected at day + 16, reaching maximum volume at day + 35. (**B**) PDX tumor on NOD scid gamma mouse (day + 35); (**C**) PDX tumor explanted

3D PDOs (UB_PCa03) were derived from fresh liver metastatic tumor tissue, following the protocol described in the Methods section, and could be successfully used for drug response assays (Fig. 6A). Por PDX derivation, 1.8 × 10^6^ viable cells (passage 6), were sc injected in an NSG male mouse. Palpable sc tumor was first detected at day + 16, reaching maximum volume at day + 35 (Fig. 6B-D).

### Drug vulnerabilities

We used the generated PDOs to assess sensitivities to several drugs of interest, including drugs commonly used in mCRPC, as well as selected experimental agents potentially targeting the Wnt/β-catenin pathway. As predicted by the patient disease course, PDOs showed lack of sensitivity to treatment with ARPI (enzalutamide), as well as chemotherapy with docetaxel. Among the experimental drugs potentially targeting the Wnt/β-catenin, we assessed response to the spindle assembly checkpoint kinase inhibitor (TTKi) CFI-402,257 [21], the casein kinase 1 (CK1) inhibitor IC261 [22], the tankyrase 1/2 inhibitor G007-LK [23, 24], the forkhead box M1 (FOXM1) inhibitor RCM1 [6], the Wnt/β-catenin inhibitor iCRT14 [25]. Due to the implications of SWI/SNF complex-targeting agents in AR-driven PCa [26] and Wnt-driven PCa [16] fxc, we included the BRD9 and BRD7 proteolysis-targeting chimera (PROTAC) degrader VZ185 [27, 28], the PROTAC degraders of SMARCA2 and SMARCA4 (subunits of the SWI/SNF complex) AU15330, A947, ACBI1, and the dual SMARCA4/SMARCA2 inhibitor FH286. Within the assayed drugs, uniquely the targeted treatment with IC261 (IC50 = 0.47 µM) and G007-LK showed a modest effect on PDOs viability (Suppl. Figure 1). For enzalutamide and docetaxel, results were compared with sensitivities of two established PCa PDOs (MSKPCa8 and PM154) (Suppl. Figure 2).

## Discussion

We report a rare transformation of a metastatic PCa, acquiring histological features of urothelial carcinoma, atypical IHC pattern and a highly aggressive disease course. However, DNA NGS from liver metastatic biopsies revealed, along with the clonal *CTNNB1* gain-of-function mutation (D32N), molecular findings characteristic for PCa, such as the presence of a *TMPRSS2* rearrangement, a loss-of-function mutation in *TP53* (R282W), and *PTEN* loss. FISH performed on PDX tumor material could confirm the presence of the *TMPRSS2: ERG* rearrangement, reassuring the final diagnosis of PCa metastases. In line with patient aggressive clinical disease course, derived PDOs showed lack of sensitivity to common drugs used for mCRPC, such as enzalutamide and docetaxel. We further assessed sensitivity to several experimental drugs with potential activity in tumors with Wnt/β-catenin pathway activation. Our preliminary drug response results showed only modest sensitivity to treatment with the CK1 inhibitor IC261 and the tankyrase inhibitor G007-LK, which are known to interfere with Wnt/β-catenin signaling in PCa [7]. Further experiments would be required to confirm these findings. CK1α phosphorylates β-catenin and induces its degradation, so that inhibition of CK1α leads to an excessive Wnt/β-catenin activation ^3,29^. Moreover, both CK1α and CK1δ phosphorylate the Wnt co-receptor low-density lipoprotein receptor-related protein 6 (LRP6), resulting again in Wnt/β-catenin pathway activation [29, 30]. IC261 inhibits CK1δ, CK1ε and CK1α, therefore, having potential impact on the WNT/β-catenin signaling. However, further work showed that IC261 may induce Wnt-independent cancer cell death, contrary to other CK1δ/ε inhibitors [22]. Tankyrase 1/2 inhibitors lead to decreased degradation of axin, a negative regulator of the Wnt/β-catenin promoting β-catenin degradation [31]. Based on previous data showing that BRG1 (SMARCA4), a key component of the SWI/SNF chromatin remodeling complex, plays a relevant role in the Wnt//β-catenin pathway regulation, we additionally aimed to assess sensitivity to SMARCA2 and SMARCA4 PROTAC degraders and inhibitors [16, 32–34]. Moreover, increased Wnt//β-catenin signaling activity has been demonstrated in PCa tumors with high SMARCA4 levels [35]. However, none of the used drugs targeting the SWI/SNF complex components SMARCA4 and SMARCA2 showed activity in the reported PDO.

Finally, the morphologic and molecular features of this double negative PCa highlight a challenge in the classification of advanced metastatic CRPC. Histology alone, unlike primary untreated PCa, is insufficient to adequately characterize these tumors. A recent working group on the pathology of CRPC is supporting a classification approach that includes morphology, molecular, and IHC information (Hafner, Rubin, Beltran, in preparation). For example, in this case high-grade adenocarcinoma would be modified with the description of double negative CRPC referring to the lack of AR activity or neuroendocrine features; these features may only become apparent through molecular testing. The working group comprised of pathologists, oncologists, and a surgeon specialized in prostate cancer, noted that this information may have important clinical treatment implications.

## Conclusions

We characterized a rare histologic transformation of an advanced *CTNNB1*-mutated mCRPC, clinically characterized by an aggressive lung and hepatic metastatic spread and fulminant disease course. We successfully derived PDOs and PDX from liver metastatic tumor material. Derived PDOs showed, as expected, lack of response to common drugs used in mCRPC, such as enzalutamide and docetaxel. Preliminary experiments showed only a modest sensitivity to the CK1 inhibitor IC261 and to the tankyrase inhibitor G007-LK, which are known to interfere with Wnt/β-catenin signaling. Further research work is needed to explore the effect of these compounds in more detail. The reported mCRPC case underlines the need of further research work required to enable successful targeted treatment of rare molecular subtypes of PCa.

### Electronic supplementary material

Below is the link to the electronic supplementary material.

Supplementary Material 1

Supplementary Material 2

## Data Availability

The datasets generated, as well as the derived PDO, during the current study are available from the corresponding author on reasonable request. NGS results from liver metastasis are provided as Suppl. Table 1.

## Abbreviations

AR: androgen receptor
ARPI: androgen receptor pathway inhibitor
CK1: casein kinase 1
ERG: ETS-related gene
FISH: fluorescent in situ hybridization
FOXM1: forkhead box M1
IHC: immunohistochemistry
mCRPC: metastatic castration-resistant prostate cancer
NGS: next-generation sequencing
NSG: NOD scid gamma
PCa: prostate cancer
PDO: patient-derived organoid
PDX: patient-derived xenograft
PROTAC: proteolysis-targeting chimera
PSA: prostate-specific antigen
PSAP: prostatic acid phosphatase
Sc: subcutaneously
