# Supplementary material for: Rare histologic transformation of a CTNNB1 (β-catenin) mutated prostate cancer with aggressive clinical course
Source: Diagn Pathol. 2024 Jun 21;19:83. doi: 10.1186/s13000-024-01511-3 (PMC11191256; doi:10.1186/s13000-024-01511-3)
Supplement: Supplementary file 2 — Supplementary Material 2 [file 13000_2024_1511_MOESM2_ESM.docx]

**Rare histologic transformation of a *CTNNB1* (β-catenin) mutated prostate cancer with aggressive clinical course**

Dilara Akhoundova^1,2^, Stefanie Fischer^3^, Joanna Triscott^1^, Marika Lehner^1^, Philip Thienger^1^, Sina Maletti^1^, Muriel Jacquet^1^, Dinda S.H. Lubis^1^, Lukas Bubendorf^4^, Wolfram Jochum^5^ and Mark Rubin^1,6^

**Supplementary Figures**

**
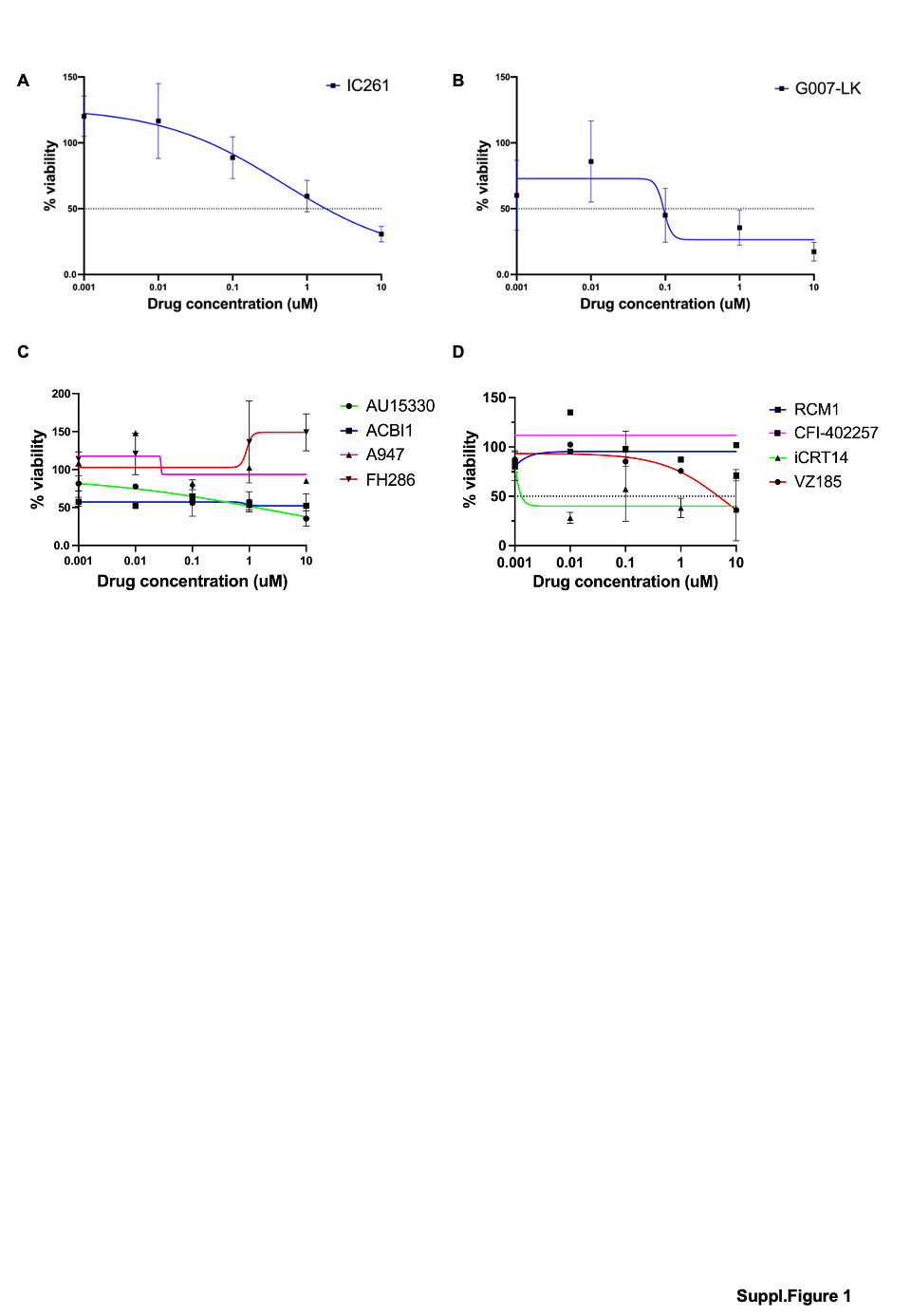
**

**Suppl. Figure 1. Drug response curves to enzalutamide and docetaxel.** (A) For the reported PDO (UB_PCa03) and (B-C) two established androgen-independent PCa PDOs: (B) MSKPCa8 (adenocarcinoma), and (C) PM154 (neuroendocrine).


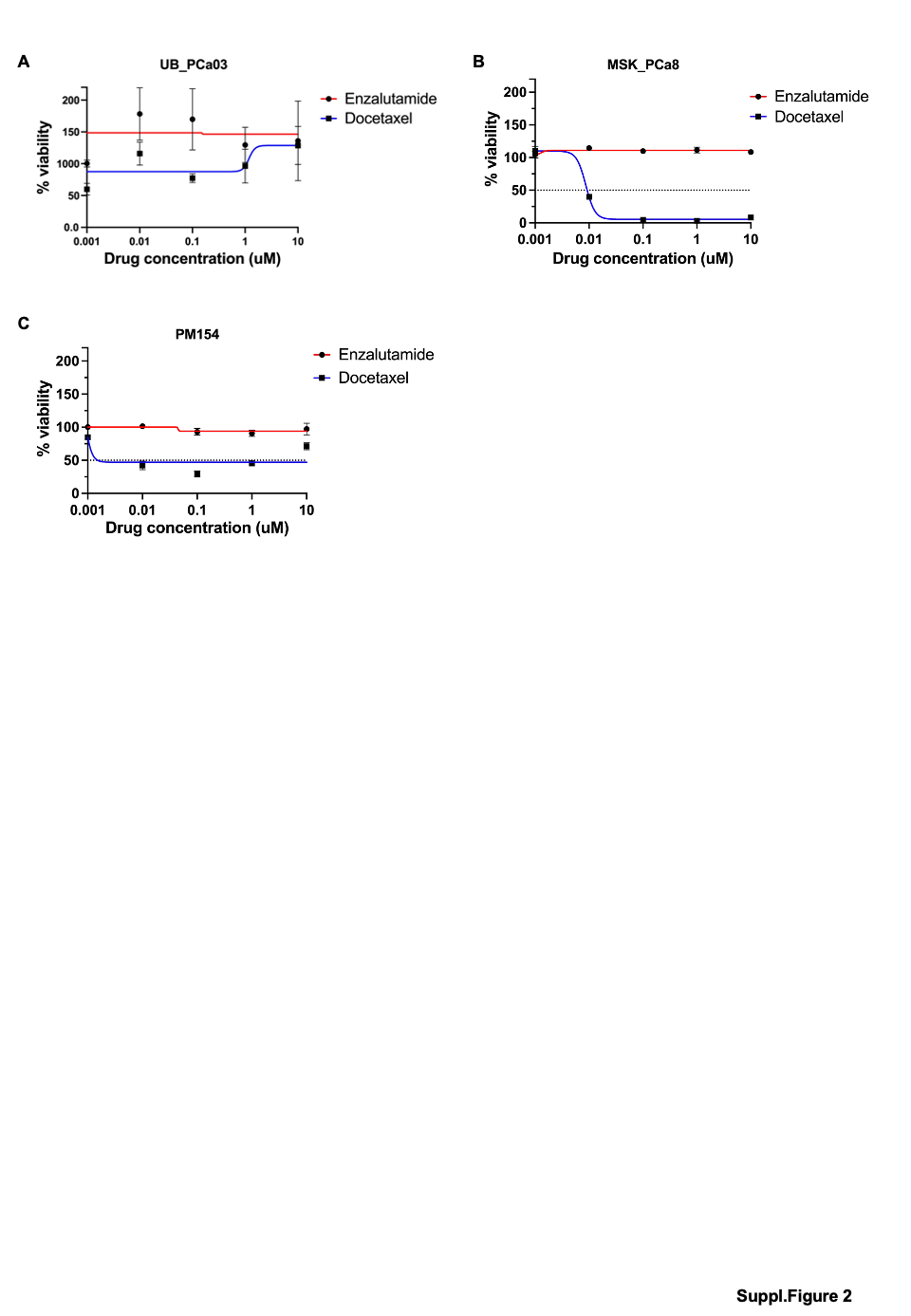
 **Suppl. Figure 2. Drug response curves to experimental drugs of interest in reported PDO (UB_PCa03).** Modest response to targeted treatment with (A) the casein kinase inhibitor IC261 (IC50= 0.47 μM) and (B) the tankyrase 1/2 inhibitor G007-LK (IC50= 0.1 μM). Lack of response to (C) the SMARCA2/4 PROTAC degraders and inhibitor (AU15330, ACBI1, A947 and FH286); as well as to (D) other assayed drugs with potential effect on Wnt/β-catenin signaling (CFI-402257, iCRT14, RCM1 and VZ185).
